# Dapagliflozin mitigates cognitive deficits in a rat model of chronic restrained stress by addressing insulin resistance and mitochondrial dysfunction

**DOI:** 10.1007/s00210-025-04136-5

**Published:** 2025-05-21

**Authors:** Nourhan M. Gamal, Wesam El Bakly, Sherin S. T. Saad, Dalia A. A. El Waseef, Amal S. El-Shal, Wessam Ezzat, Yosra M. Magdy

**Affiliations:** 1 Clinical Pharmacology Department, Faculty of Medicine, Ain-Shams University, Cairo, Egypt; 2 Clinical Pharmacology Department, Armed Forces College of Medicine, Cairo, Egypt; 3 Histology Department, Faculty of Medicine, Ain-Shams University, Cairo, Egypt; 4 Medical Biochemistry and Molecular Biology Department, Faculty of Medicine, Zagazig University, Zagazig, Egypt; 5 Medical Biochemistry and Molecular Biology Department, Armed Forces College of Medicine, Cairo, Egypt; 6 Physiology Department, Faculty of Medicine, Ain-Shams University, Cairo, Egypt; 7 Physiology Department, Armed Forces College of Medicine, Cairo, Egypt

**Keywords:** Chronic restrained stress, Cognitive impairment, Dapagliflozin, Oxidative stress, Mitochondrial dysfunction, Insulin resistance

## Abstract

Chronic stress is recognized as a risk factor for neurodegeneration. Sodium glucose co-transporter 2 receptors (SGLT2) have been found in various brain regions, suggesting the potential neuroprotective properties of SGLT2 inhibitors as dapagliflozin (DGF). This study aimed to investigate the effect of DGF on behavioral, and neurodegenerative changes in chronic restraint stress (CRS) as an animal model of cognitive impairment. Forty-eight male rats were allocated into four groups: *Control*; *CRS-subjected group*, rats were subjected to chronic restraint stress for 6 weeks to induce cognitive impairment; *DGF-treated CRS group*, dapagliflozin was given daily by oral gavage; and *DGF-administered group*. Behavioral tests were performed and fasting serum glucose, insulin, and corticosterone levels were measured*.* Hippocampal oxidative markers, insulin signaling, mitochondrial function, amyloid beta, p-tau, and brain-derived neurotrophic factor (BDNF) gene expression were evaluated. DGF significantly prevented CRS-induced cognitive dysfunction (Y maze and Morris water maze tests). Also, DGF ameliorated hippocampal neurodegenerative changes by decreasing tau and amyloid beta levels, while increasing BDNF gene expression. DGF reduced hippocampal phosphorylated mammalian target of rapamycin (p-mTOR) and protein kinase B (p-Akt) levels. In addition to its antioxidant effects, DGF increased ATP levels and cytochrome C oxidase activity. These findings were confirmed by transmission electron microscopic (TEM) examination. The current study demonstrates a biological link between chronic stress, insulin resistance, and cognitive impairment. Dapagliflozin has therapeutic potential in alleviating cognitive deficits and neurodegeneration primarily due to its insulin-sensitizing and antioxidant properties, along with its capacity to enhance mitochondrial function.

## Introduction

Chronic stress can lead to functional and structural changes in the brain, impacting cognitive functions such as learning and memory. This makes chronic stress a significant risk factor for AD (Saeedi and Rashidy-Pour 2021). Stress activates glucocorticoid receptors in the hippocampus and decreases neuronal cell survival and neurogenesis (Ryu et al. 2016). Further, stress reduces the hippocampal protein expression of brain-derived neurotrophic factor (BDNF) (Zhang et al. 2017) which has a crucial role in active dependent synaptic plasticity and neuronal survival in adult brain (Lepack et al. 2014) resulting in impairment of memory and cognition (Radahmadi et al. 2015).

Type 2 diabetes is a well-known risk factor for cognitive impairment and dementia through impairment of insulin sensitivity, accumulation of Aβ and hyper-phosphorylation of tau protein (Tumminia et al. 2018). Recent evidence suggests that insulin plays a role in enhancing synaptic viability, dendritic spine formation, and neurotransmitter turnover. Insulin also can affect the clearance of Aβ peptides and the phosphorylation of tau, which are characteristic features of AD. Additionally, insulin influences vascular function through its effects on vasoreactivity, lipid metabolism, and inflammation. Dysregulation of insulin function can potentially contribute to neurodegeneration through these various pathways (Kellar and Craft 2020). One important downstream pathway of insulin signaling involves the binding of phosphatidylinositol 3-kinase (PI3 K) to activated insulin receptor substrate (IRS) proteins. PI3 K activation leads to the activation of Akt kinase, which plays a critical role in promoting cell survival. Akt, in turn, activates the mammalian target of rapamycin (mTOR), a protein that regulates protein synthesis and inhibits autophagy. Persistent activation of mTOR, as seen in chronic conditions, may contribute to metabolic dysfunction that leads to the breakdown of the blood–brain barrier, hyperphosphorylation of tau protein, and the formation of senile plaques (Cai et al. 2015).

Sodium-glucose co-transporter 2 inhibitors (SGLT2i) are oral medications used to treat type 2 diabetes mellitus. SGLT2 is primarily expressed in segments 1 and 2 of the proximal convoluted tubule in the kidney and plays a crucial role in the reabsorption of glucose from urine by utilizing a sodium concentration gradient (Nauck 2014).

While SGLTs were initially thought to be primarily expressed in the kidneys, emerging evidence suggests their presence in the central nervous system of mammals. Research has demonstrated the expression of the SGLT1 receptor in areas such as CA1, CA3 (regions 1 and 3 of the hippocampal cornu ammonis), the dentate gyrus hippocampal subfields, cerebellum, and the endothelial cells of the blood–brain barrier (Jurcovicova et al. 2014). This specific distribution of SGLTs in the brain may be responsible for the intriguing evidence suggesting their potential neuroprotective properties. Recent studies have declared a potential relationship between SGLT2 inhibitors and AD. They have shown effectiveness in reducing senile plaque density and insoluble amyloid β levels in the cortex and hippocampus of a murine model of AD and type 2 diabetes (Hierro-Bujalance et al. 2020). This suggests their potential therapeutic role in reducing AD-related pathology. Dapagliflozin (DGF), a SGLT2 inhibitor, has been reported to have beneficial effects on cognitive impairment in a rat model of scopolamine-induced memory impairment via its acetylcholinesterase inhibiting activity, which is an additional property of SGLT inhibitors (Rizvi et al.^2014^ and Arafa et al.^2017^).

These findings provide preliminary evidence for the potential neuroprotective and cognitive-enhancing effects of SGLT2 inhibitors in the context of AD and cognitive impairment. Therefore, the study was designed to investigate the effects of dapagliflozin on cognitive deficits, insulin resistance, and mitochondrial dysfunction in the hippocampus of rats subjected to chronic restrained stress.

## Materials and methods

### Drugs and chemicals

Dapagliflozin propranediol monohydrate *(Sunshine organics PVT. Ltd)* was supplied as off-white to yellow solid crystalline powder and dissolved in dimethylsalfoxide 0.1% (DMSO) (*SDFCL, India*).

### Experimental animals

Forty-eight male Albino Wistar rats weighing 150–200 g were purchased from the National Research Institute (Cairo, Egypt). This research was conducted in The Medical Research Center and Department of Pharmacology at the Faculty of Medicine at Ain Shams University. Rats were housed under standard conditions (12-h light/dark cycle, temperature of 23 ± 2 °C), in plastic cages (4 rats/cage) with free access to water and food. They were fed standard rat chow (Meladco for Animal Food, Egypt). Rat chow pellets typically contain 20% protein, 10% fat, and 70% carbs. All procedures involving animals followed the rules of the Institutional Animal Ethics Committee for Ain Shams University, Faculty of Medicine (FMASU MS 367/2022).

### Experimental procedures

The study period was 6 weeks. Rats were randomly allocated in four equal groups (12 rat per group) as follows: *Control group* (rats received DMSO 0.1% in 1 ml saline by oral gavage for 6 weeks), *CRS-subjected group* (rats were subjected to CRS and received DMSO 0.1% in 1 ml saline by oral gavage for 6 weeks), *DGF-treated CRS group* (rats were subjected to CRS for 6 weeks and simultaneously received dapagliflozin, 1 mg/kg/day by oral gavage for 6 weeks) and *DGF-administered group* (rats received dapagliflozin only, 1 mg/kg/day for 6 weeks).

#### Drug intervention

DGF was administered chronically throughout the 6 weeks of the experiment. It was given daily in a dose of 1 mg/kg (Arab et al. 2021) dissolved in DMSO 0.1%, adjusted that every rat received 1 ml by oral gavage. Rats in the control group were received equal amount of DMSO 0.1% by oral gavage at the same time point.

#### Induction of chronic restrained stress

Animals were restrained to induce cognitive impairment by placing rats in plexiglass tubes with dimensions 25 cm × 8 cm, wide enough to allow comfortable breathing but restricting their movement. Every stress session was carried out between 09:00 a.m. and 1:00 p.m. at fixed time daily to avoid the effects of changes in circadian rhythm for 4 h daily for 6 weeks; the movement of the animal was restricted totally (Bravo et al. 2009). Out of these restraint stress sessions, rats were housed in groups of 4 per cage. Rats were weighed at the beginning of the study and weekly till the end of the study using the 3-digit electronic balance Precisa, Switzerland (in grams and was expressed as an absolute value).

## Behavioral tests

All rats underwent the following behavioral assessments at the end of the experiment (fourth week): By using the Y Maze spontaneous alternation test, for evaluation of short-term memory then the Morris Water Maze was used to assess cognitive ability and spatial memory. Each behavioral test was administered 2 days apart. The next morning animals were sacrificed.

### Y maze spontaneous alternation test

Y maze test is used to evaluate short-term memory in rats. By permitting rats to explore all three arms of the maze, one can test rat’s spontaneous alternation, a measurement of spatial working memory that is motivated by rodents’ natural eagerness to explore previously unexplored locations. If the rat has an intact working memory and intact prefrontal cortex, it will recall the arms that have been visited in the past and display a propensity to enter a less recently visited arm (Sarnyai et al. 2000). One entry means that the rat has its four legs become inside one arm of the maze either A, B, or C. While alternation means the rat enters all the three arms successively. A superimpose technique is used to recognize spontaneous alterations. A manner of A–B–C–B–A consists of two alternations, as an example. The percent (%) alternation was calculated using the following formula (Nasri et al. 2012):

  

$$\%\;\mathrm{Alternation}\hspace{0.17em}=\hspace{0.17em}\mathrm{Number}\;\mathrm{of}\;\mathrm{Alternations}/(\mathrm{Total}\;\mathrm{number}\;\mathrm{of}\;\mathrm{armentries}-2)\times100.$$

### Morris water maze test

It is a very sensitive and well-validated measure of rodent cognitive function. It is the rodent’s standard test for spatial memory. This task is severely impacted by hippocampus lesions that only affect 30–50% of the total hippocampal volume (Broadbent and Thomson 2006).

Rats underwent four trials in the Morris water-maze on each of 5 days as part of a spatial training program (Anisman and Mcintyre 2002). The submerged platform was stationary in one corner of the maze during testing, and the latency to locate it was calculated. Each trial in this experiment and the ones that followed involved carefully placing a single rat into the water, facing the pool’s edge, at one of four potential beginning sites (e.g., north, south, east, and west). Each trial’s starting site was randomly selected provided that all start sites were used on every given day. When the rat arrived at the platform and stayed there for 10 s, the trial came to an end, and the latency was recorded. The trial was stopped, and the rat was left on the platform for 10 s if it took the rat longer than 120 s to reach the platform. Rats were then moved to a dry waiting cage, where they remained for 60 s prior to the subsequent experiment. Rats were put back into their home cages after training. Rats underwent an additional 60-s probe experiment on the 6 th day without a platform in the pool. As before, rats were dropped into the pool, and both the time spent inside a platform quadrant and the latency to reach the target quadrant were recorded.

On the day of sacrifice, rats were anesthetized with an intraperitoneal injection of urethane at a dose of 1.2 g/kg. Blood samples were collected and centrifuged at 3000 g for 10 min to obtain sera, which were then stored at − 80 °C until biochemical analysis. The hippocampal tissues were carefully dissected out for histopathological examination. These tissues were fixed in 10% formalin, embedded in paraffin, and sectioned at 5-µm thickness. The sections were stained with hematoxylin and eosin (H&E) to assess general morphology.

## Biochemical studies

### Assessment of glucose homeostasis

Fasting serum glucose was measured by automated spectrophotometric method using Synchron cx5 autoanalyzer (*Sigma-Aldrich, Inc.*,* USA*). Fasting serum insulin level was measured using rat insulin ELISA kit (*RayBio® ELR-Insulin*), according to manufacturer’s instructions. Insulin resistance (*IR*) was calculated using the following formula (Matthews et al.^1985^).

  

$$\mathrm{HOMA}-\mathrm{IR}\hspace{0.17em}=\hspace{0.17em}\mathrm{fasting}\;\mathrm{blood}\;\mathrm{glucose}\;(\mathrm{FBG})(\mathrm{mmol}/\mathrm L)\hspace{0.17em}\times\hspace{0.17em}\mathrm{fasting}\;\mathrm{blood}\;\mathrm{insulin}\;(\mathrm{FBI})(\mu\mathrm{IU}/\mathrm{ml})/22.5(\mathrm{HOMA}-\mathrm{IR}\hspace{0.17em}\geq\hspace{0.17em}2.8\;\mathrm{represents}\;\mathrm{insulin}\;\mathrm{resistance}\;\mathrm{state}).$$

### Determination of hippocampal oxidant/antioxidant markers

Rat malondialdehyde (MDA) ELISA kit (*Lifespan Bioscience, Inc., North America*) and rat reduced glutathione (GSH) ELISA kit (*Shanghai Blue Gene Biotech Co., Ltd)* were used for quantitative determination of hippocampal MDA and GSH concentrations, respectively. The procedure was done according to manufacturer’s instructions.

### Determination of hippocampal insulin signaling (pAKT and mTOR)

Rat phosphorylation AKT ELISA (*Biocompare, USA*) and rat p-mTOR ELISA kit (*Biocompare, USA*) were used for the quantitative determination of hippocampal pAKT and mTOR concentrations. The procedure was done according to manufacturer’s instructions.

### Assessment of mitochondrial function

Hippocampal ATP content and cytochrome C oxidase (CCO) concentration were measured using ATP ELISA kit (*Abbexa Ltd*) and rat CCO ELISA kit (*Biocompare, USA*), respectively. The procedure was done according to manufacturer’s instructions.

### Determination of hippocampal amyloid beta (Aβ1–42) and p-tau proteins

Rat Aβ1–42 ELISA kit (*MyBiosource, USA*) and p-tau ELISA kits ***(Biocompare, USA***) were used to detect Aβ1 ~ 42 and the phosphorylated form of protein tau in tissue homogenate. The procedure was done according to manufacturer’s instructions.

### BDNF mRNA expression

Total RNA was extracted from brain tissue homogenate using the RNeasy extraction kit (Qiagen). The cDNA was obtained from extracted total RNA (5 μg) with 1 μl (20 pmol) antisense primer and 0.8 μl superscript AMV reverse transcriptase for 60 min at 37 °C. The relative expression of mRNA was measured using SYBR Green method by Applied Biosystems. The concentration and purity of RNA were determined with an ultraviolet spectrophotometer. The housekeeping gene, GADPH, was used as the internal reference gene. The BDNF forward primer sequence: 5′-GTCCCTTCTACACTTACCTCTTG- 3′; the BDNF reverse primer sequence: 5′-CTTTGTTTCACCCTTTCCACTCCT- 3′. The GAPDH forward primer sequence: 5′-CACGGCAAGTTCAACGGCACAG- 3′; the GAPDH reverse primer sequence: 5′-ACGCCAGTAGACTCCACGACAT- 3′. Quantitative real-time PCR (qPCR) was performed in a total 25 μl reaction volume consisting of 2 × SYBR Green PCR Master Mix (Applied Biosystems), 900 nM of each primer, and 3 μl of cDNA. PCR reaction conditions: denaturation at 94 °C for 5–10 min, denaturation at 94 °C for 30 s–2 min, annealing for 30 s–2 min at 72 °C, extension for 5 min at 72 °C, and extension for 5–10 min. The three middle steps were repeated 30 times according to the method of Guo et al. (2017). The relative gene expression was calculated by normalizations of each target gene caspase 3, caspase 8, and caspase 9 to β-actin gene (house-keeping gene) by comparative Ct method (Schmittgen and Livak 2008).

## Histopathological study of hippocampus

The brain samples were collected (5 rats from each group); each brain was divided sagittally into two parts, one half was processed for light microscopic study. It was placed in 10% formalin for fixation. After fixation, dehydration was done in ascending grades of ethyl alcohol, followed by clearing in xylene and embedding in paraffin wax. Paraffin Sects. 4–6-µm thickness were obtained. They were stained by the following stains:

1. Hematoxylin and eosin (H & E)
  The thickness of the pyramidal layer of the CA1 region of the hippocampus in antro-posterior brain sections, and the number of degenerated cells in the CA1 region of the hippocampus in antro-posterior brain sections were estimated using an image analyzer program. The hippocampus in rats is a distinct and well-defined structure that can be easily recognized once the brain is extracted and placed under proper lighting and magnification. Because of its shape and location, careful dissection was sufficient to isolate it without the need for stereotaxic guidance (Spijker 2011). The image analyzer Leica Q win V.3 program installed on a computer in the Histology Department, Faculty of Medicine, Ain Shams University, was used. The computer was connected to a Leica DM2500 microscope (Wetzlar, Germany). Five specimens from five different rats of each group were examined. For each specimen, five non-overlapping high-power fields (HPF) were used to measure the following:
  - *The thickness of pyramidal layer of the hippocampus in H&E-stained sections*: The thickness of pyramidal cell layer measured in micrometers (μm) in the CA1 region of the hippocampus in antro-posterior brain sections was measured using magnification lens (× 10).
  - *Number of damaged pyramidal cells/HPF in the hippocampus, in H&E-stained sections: *Pyramidal cells with rounded nuclei and visible nucleoli were considered undamaged, while darkly stained shrunken cells were considered damaged neurons. The number of damaged pyramidal cells/HPF (using magnification lens × 20) in the CA1 region of hippocampus in antro-posterior brain sections was counted in H&E-stained sections from each group.
2. Iron hematoxylin stain (Iron Hx) for detection of mitochondria
  The area % of the deeply stained perinuclear area in the pyramidal cells of CA1 region of the hippocampus in antro-posterior brain sections (area representing mitochondria) was measured in Iron Hx-stained sections (using magnification lens × 20).

### Transmission electron microscopic study (TEM)

The hippocampus was dissected from the other half of each brain sample, cut into small pieces (1 × 1 mm) and immediately fixed in glutaraldehyde. Specimens were then rinsed in phosphate buffer and post fixed in 1% osmic acid. Dehydration was done in ascending grades of alcohol followed by clearing in propylene oxide, then embedding in epon. Semi-thin sections were cut at 1 µm and stained with 1% toluidine blue; they were used for orientation. Ultra-thin sections of 80 nm in thickness were obtained from the selected areas (CA1). They were picked up on copper grids and then stained and examined using the transmission electron microscope JEOL JEM 1010 in the EM unit, Faculty of Science, Ain Shams University.

## Statistical analysis

All data were expressed as *mean* ± *SD*. Statistical analysis was carried out using Graphpad prism, software program, version 8.0 (2007) (Inc., CA, USA). Statistical difference among groups were determined using one-way analysis of variance (ANOVA) followed by post hoc Tukey’s multiple comparison test. But for body weight and Morris water maze, two-way ANOVA followed by post hoc Tukey’s multiple comparison test were used.

## Results

### The effect of DGF on body weight changes in CRS-induced cognitive impairment in male Wistar rats

**Fig. 1:**
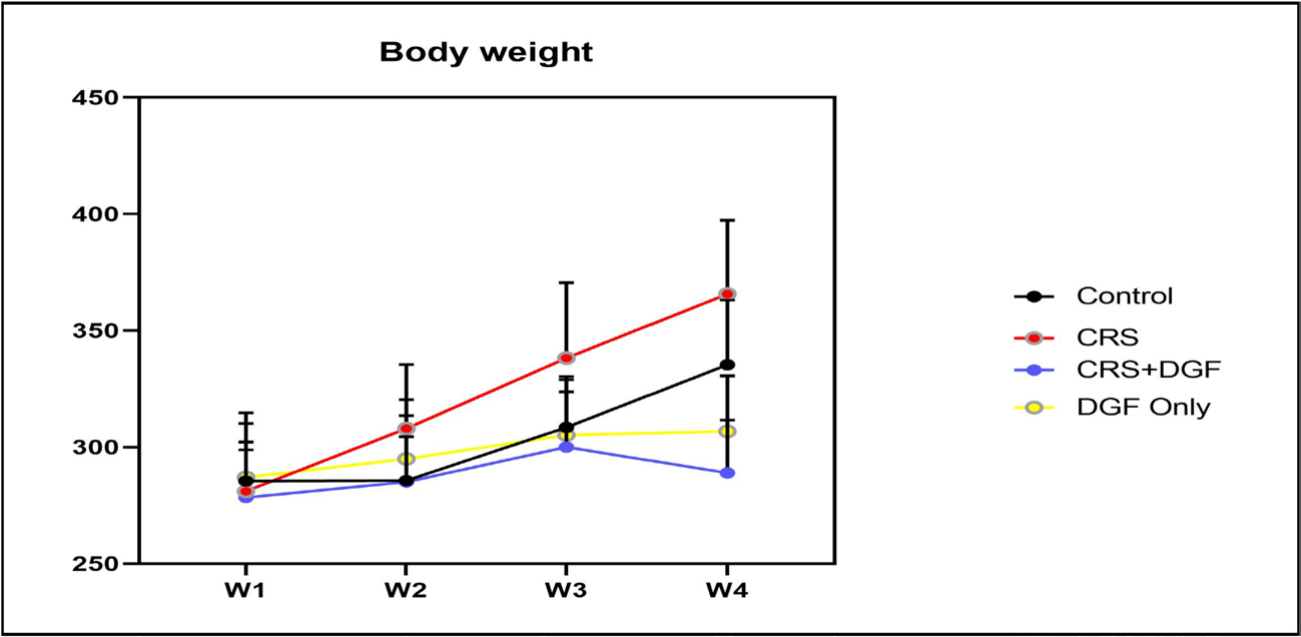
Effect of DGF treatment on body weight in chronic restraint stressed male rats. CRS, chronic restraint stress group; CRS + DGF, chronic restraint stress plus DGF administered. * Significantly different from control group, # significantly different from treated group respectively at *P* < 0.05 using two-way ANOVA test followed by Tukey’s post hoc multiple comparison test. Data are expressed as *mean* ± *SD* (*n* = 12)

Body weight showed no significant change between the four groups (control, CRS, CRS + DGF, and DGF Only) in week 1 and week 2. However, the body weight increased significantly (*P* < 0.01) to 12.6% and 26.6%, respectively in CRS group compared to CRS + DGF group in weeks 3 and 4, respectively (Fig. 1).

### The effect of DGF on memory and learning impairment assessed in CRS induced cognitive impairment in male Wistar rats

Cognitive impairment induced by chronic restraint stress of Wistar male rats for 6 weeks significantly decreased the spontaneous alteration ratio in Y maze test (*P* < 0.0001), significantly increased the latency to find the platform in Morris water maze during training on days 3, 4, and 5 (*P* < 0.0001), significantly increased the latency to find the platform (*P* < 0.0001) on the 6 th day, significantly decreased the time spent inside the platform quadrant in Morris water maze test (*P* < 0.0001) to 0.3-fold in CRS group as compared to control group.

**Fig. 2:**
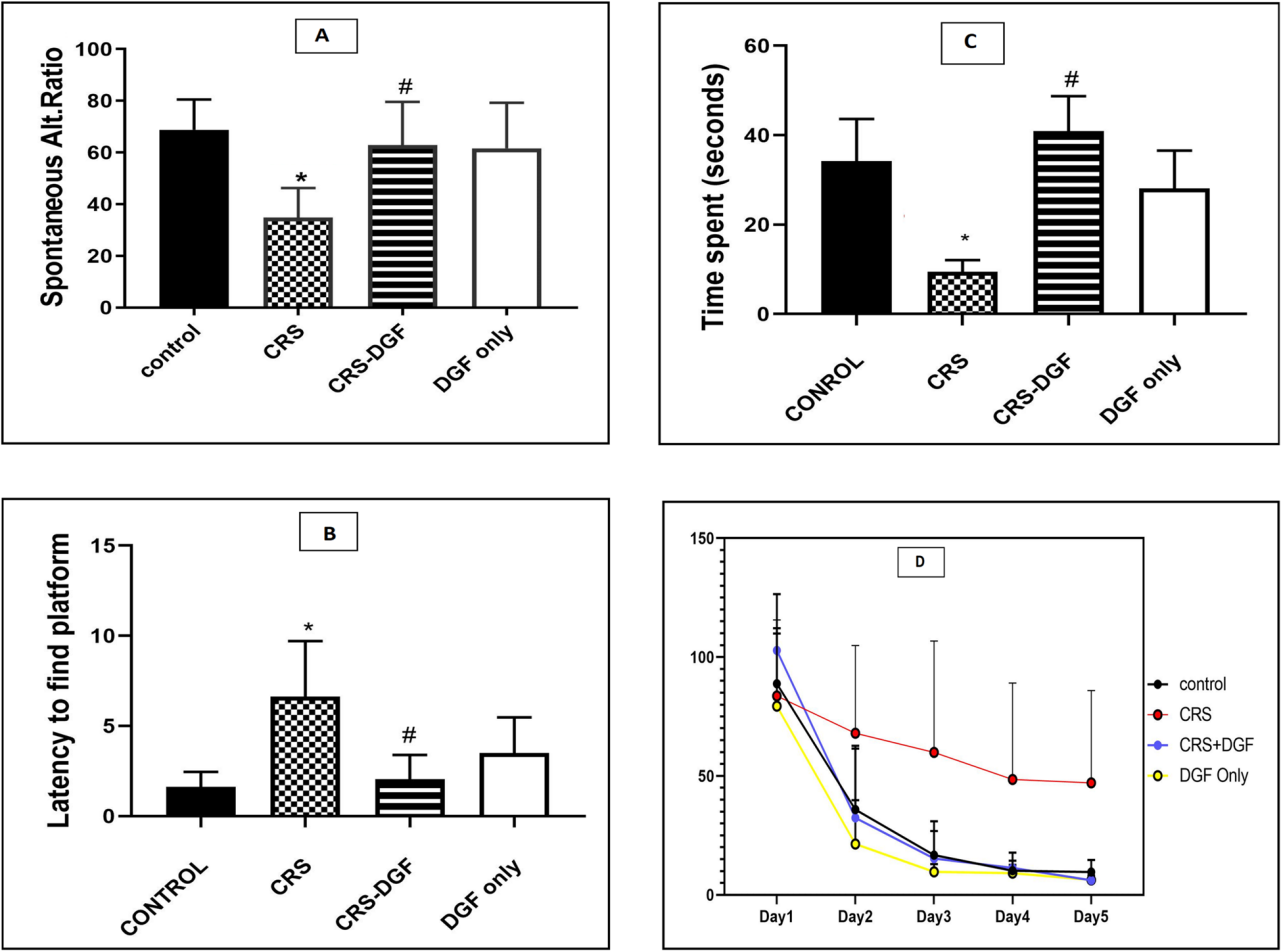
Effect of DGF treatment on spontaneous alteration ratio by Y maze test (legend **A**), on latency to find platform assessed by Morris water maze test (legends **B** and **D**) and on the time spent inside the platform quadrant on day 6 assessed by Morris water maze (legend **C**) in chronic restraint stressed male rats. *CRS, chronic restraint stress group; CRS* + DGF, chronic restraint stress plus DGF administered. * Significantly different from control group, # significantly different from treated group respectively at P < 0.05 using two-way ANOVA test, followed by Tukey’s post hoc* multiple comparison test. Data are expressed as mean* ± *SD (n* = *12)*

DGF treatment significantly increased the spontaneous alteration ratio in CRS-DGF administered group (*P* < 0.0001), significantly decreased the latency to find the platform during training on days 3, 4, and 5 (*P* < 0.001) significantly decreased the latency to find the platform significantly on the 6 th day (*P* < 0.0001), significantly increased the time spent inside the platform quadrant (*P* < 0.0001) to as compared to the CRS group (Fig. 2a, b, c, and d).

### The effect of DGF on fasting serum glucose, fasting serum insulin, and HOMA IR index in CRS-induced cognitive impairment in male Wistar rats

Chronic restraint stress significantly increased fasting serum glucose, fasting serum insulin levels, and HOMA IR index (*P* < 0.01) as compared to control group, while DGF treatment significantly decreased fasting glucose serum level and HOMA IR index (*P* < 0.01) as compared to the CRS group (Table 1).

Chronic restraint stress of Wistar male rats significantly increased serum corticosterone level (*P* < 0.0001) as compared to control group, while DGF treatment in CRS-DGF-administered group significantly decreased serum corticosterone level (*P* < 0.001) as compared to the CRS group (Table 1).

**Table 1:** Serum levels of fasting glucose, insulin, HOMA-IR index, and serum corticosterone levels in the different studied groups

	Fasting serum glucose level (MMOL/l)	Fasting serum insulin level (UIU/ml)	HOMA-IR index	Serum corticosterone level (ng/ml)
Control	5.07 ± 0.60	8.20 ± 0.70	1.84 ± 0.21	29.03 ± 6.33
CRS	19.80* ± 4.40	16.93* ± 11.65	15.10* ± 5.37	108.20* ± 8.71
CRS + DGF	11.27^#^ ± 1.60	11.65 ± 1.68	5.76^#^ ± 0.31	65.70^#^ ± 13.29
DGF Only	4.90 ± 0.30	7.84 ± 0.21	1.70 ± 0.11	29.43 ± 4.33

*CRS* chronic restraint stress group, *CRS* + *DGF* chronic restraint stress plus DGF administered

* Significantly different from control group, # significantly different from treated group, $ CRS + DGF significantly different from control group, respectively, at *P* < 0.05 using one-way ANOVA test followed by Tukey’s post hoc multiple comparison test. Data are expressed as *mean* ± *SD* (*n* = 12)

### The effect of DGF on hippocampal levels of oxidative stress markers; malondialdehyde (MDA) and reduced glutathione (GSH) in CRS induced cognitive impairment in male Wistar rats

Chronic restraint stress of Wistar male rats significantly increased hippocampal MDA level (*P* < 0.01), decreased GSH level (*P* < 0.0001) as compared to control group, while DGF treatment in CRS-DGF administered group significantly decreased hippocampal MDA level and increased hippocampal GSH level (*P* < 0.001) as compared to the CRS group (Table 2).

**Table 2:** Hippocampal tissue levels of oxidative stress markers malondialdehyde (MDA) and reduced glutathione (GSH), ATP content, and cytochrome C oxidase (CCO) in the different studied groups

	Hippocampal MDA levels	Hippocampal GSH levels	Hippocampal ATP content	Hippocampal CCO enzyme
	(nmol/mg protein)	(nmol/mg protein)	(ng/mg protein)	(ng/mg protein)
Control	58.90 ± 5.94	155.60 ± 4.31	124.70 ± 1.49	9.13 ± 1.46
CRS	168.50* ± 19.66	73.70* ± 2.26	59.23* ± 10.30	3.51* ± 1.32
CRS + DGF	88.85^#^ ± 13.22	125.50^#^ ± 4.03	111.80^#^ ± 5.38	7.17^#^ ± 1.41
DGF Only	56.75 ± 2.19	160.40 ± 1.49	126.70 ± 7.42	9.70 ± 1.15

*CRS* chronic restraint stress group, *CRS* + *DGF* chronic restraint stress plus DGF administered

* Significantly different from control group, # significantly different from treated group, $ CRS + DGF significantly different from control group, respectively, at *P* < 0.05 using one-way ANOVA test, followed by Tukey’s post hoc multiple comparison test. Data are expressed as *mean* ± *SD* (*n* = 12)

### The effect of DGF on hippocampal mitochondrial function; ATP content and cytochrome C oxidase (CCO) in CRS induced cognitive impairment in male Wistar rats

Chronic restraint stress of Wistar male rats significantly decreased hippocampal ATP content (*P* < 0.0001) and decreased CCO enzyme in the brain (*P* < 0.01) as compared to control group, while DGF treatment in CRS-DGF-administered group significantly increased hippocampal ATP content (*P* < 0.0001) and increased CCO enzyme (*P* < 0.01) as compared to the CRS (Table 2).

### The effect of DGF on hippocampal levels of protein kinase B (p-Akt) and phosphorylated mammalian target of rapamycin (p-mTOR) levels in CRS induced cognitive impairment in male Wistar rats

**Fig. 3:**
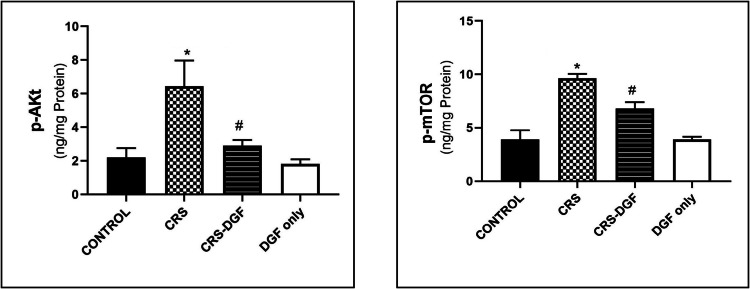
Effect of DGF treatment on hippocampal protein kinase B (p-Akt) and phosphorylated mammalian target of rapamycin (p-mTOR) levels in chronic restraint stressed male rats. *CRS, chronic restraint stress group; CRS* + *DGF, chronic restraint stress plus DGF administered.* * Significantly different from control group, # significantly different from treated group respectively at *P* < 0.05 using two-way ANOVA test, followed by Tukey’s post hoc multiple comparison test. Data are expressed as *mean* ± *SD* (*n* = 12)

Chronic restraint stress of Wistar male rats significantly increased hippocampal p-Akt, hippocampal p-mTOR levels (*P* < 0.001) as compared to control group, while DGF treatment in CRS-DGF-administered group decreased hippocampal p-Akt level significantly (*P* < 0.01) and decreased hippocampal p-mTOR level significantly (*P* < 0.001) as compared to the CRS group (Fig. 3).

### The effect of DGF on hippocampal amyloid beta, p-tau levels and hippocampal BDNF gene expression in CRS-induced cognitive impairment in male Wistar rats

**Fig. 4:**
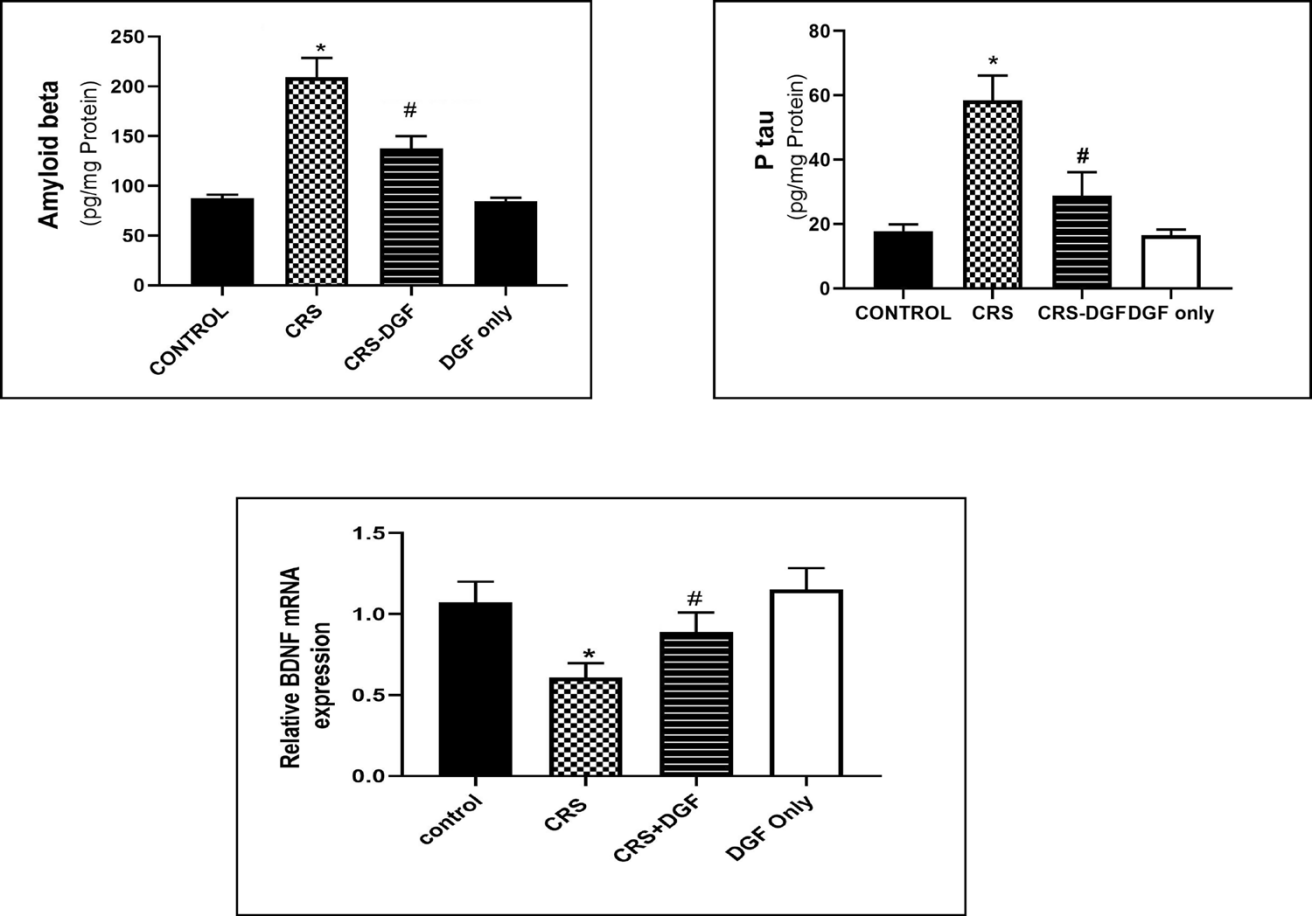
Effect of DGF treatment on brain levels of amyloid beta & p-tau and relative hippocampal BDNF mRNA expression in chronic restraint stressed male rats

Chronic restraint stress of Wistar male rats significantly increased amyloid beta, p-tau levels in the brain (*P* < 0.0001), decreased BDNF gene expression in the hippocampus (*P* < 0.0001) as compared to control group, while DGF treatment in CRS-DGF-administered group significantly decreased in amyloid beta and brain p-tau levels (*P* < 0.001), significantly increased BDNF gene expression (*P* < 0.0001) as compared to the CRS group (Fig. 4).

### Histopathological findings of hippocampal tissue

**Fig. 5:**
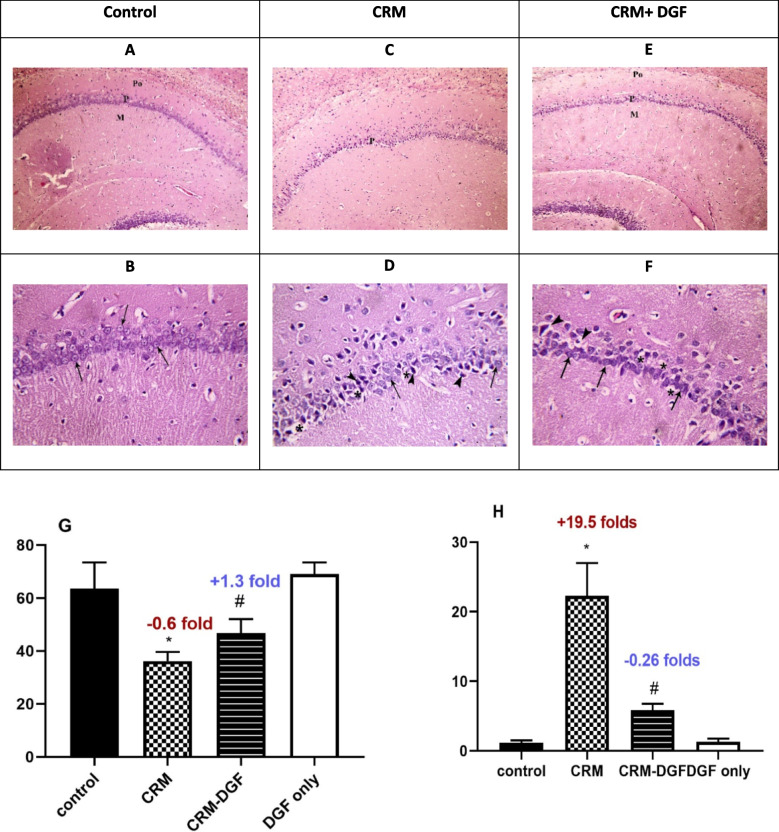
**A** and **B** Control group; **C** and **D** CRM group; **E** and **F** CRM + DGF group. **A** A photomicrograph of a section in the hippocampus of a rat in the control group showing the three layers forming the hippocampus proper; molecular (M), pyramidal (P), and polymorphic (Po) cell layers. **B** A photomicrograph of a section in the hippocampus of a rat in the control group. The pyramidal layer is formed of crowded, evenly arranged large pyramidal cells with little neuropil in between. Most of the pyramidal cells are nearly of the same size. Each cell contains a single, large, rounded, central, vesicular nucleus with prominent nucleolus (↑). **C** A photomicrograph of a section in the hippocampus of a rat in the CRM group showing irregularity and apparent thinning of the pyramidal layer (P). **D** A photomicrograph of a section in the hippocampus of a rat in the CRM group. The pyramidal cells are widely separated, irregularly arranged, and with wide, vacuolated neuropil in between (*). Many pyramidal cells are shrunken, deeply stained, and variable in size with condensed dark nuclei (filled triangle). Few pyramidal cells are seen with large, rounded, central, vesicular nuclei, and prominent nucleoli (↑). **E** A photomicrograph of a section in the hippocampus of a rat in the CRM + DGF group showing the three layers of the hippocampus proper; molecular (M), pyramidal (P), and polymorphic (Po) cell layers. Notice the mild thinning of the pyramidal layer. **F** A photomicrograph of a section in the hippocampus of a rat in the CRM + DGF, group showing slight irregularity in the arrangement of the pyramidal cells. Most of the pyramidal cells have large, rounded, central, vesicular nuclei with prominent nucleoli (↑). Few pyramidal cells appear shrunken, deeply stained, and surrounded by a clear hallow (filled triangle). The neuropil in between shows many vacuoles (*). **G** CA1 pyramidal layer thickness, chronic restraint stress of Wistar male rats significantly (*P* < 0.0001) decreased CA1 pyramidal layer thickness to 0.6-fold as compared to control group, while DGF treatment in CRS-DGF-administered group significantly (*P* < 0.0001) increased CA1 pyramidal layer thickness by 1.3-fold, and **H** degenerated cell count, chronic restraint stress of Wistar male rats significantly (*P* < 0.0001) increased degenerated cell counts to 19.5-folds as compared to control group, while DGF treatment in CRS-DGF-administered group significantly (*P* < 0.0001) decreased degenerated cell counts to 0.26-fold compared to the CRS group. **A**, **C**, and **E**: H&E × 100. **B**, **D**, and **F**: H&E × 400

H&E-stained sections of the hippocampus of rats in the *control group* and *DGF only group* showed the hippocampus proper and the dentate gyrus (D). The hippocampus proper was seen consisting of four regions: CA1, CA2, CA3, and CA4. The three layers forming the hippocampus proper are molecular, pyramidal, and polymorphic cell layers. The pyramidal layer is formed of crowded, evenly arranged large pyramidal cells with little neuropil in between. Most of the pyramidal cells are nearly of the same size. Each cell contains a single, large, rounded, central, vesicular nucleus with prominent nucleolus (Fig. 5A, B).

*Chronic restrained stress group* showed irregularity and apparent thinning of the pyramidal layer. The pyramidal cells are widely separated, irregularly arranged, and with wide, vacuolated neuropil in between. Many pyramidal cells are shrunken, deeply stained, and variable in size with condensed dark nuclei. Few pyramidal cells are seen with large, rounded, central, vesicular nuclei and prominent nucleoli (Fig. 5C, D). CRM + DGF group showed the three layers of the hippocampus proper; molecular, pyramidal, and polymorphic cell layers. The pyramidal layer shows mild thinning and irregularity in the arrangement of its cells as compared to the control group. Most of the pyramidal cells have large, rounded, central, vesicular nuclei with prominent nucleoli. Few pyramidal cells appear shrunken, deeply stained, and surrounded by a clear hallow. The neuropil in between shows many vacuoles (Fig. 5E, F).

Chronic restraint stress of Wistar male rats significantly (*P* < 0.0001) decreased CA1 pyramidal layer thickness by 43% and increased degenerated cell counts to 92 folds as compared to control group, while DGF treatment in CRS-DGF-administered group significantly (*P* < 0.0001) increased CA1 pyramidal layer thickness by 29.5% and decreased degenerated cell counts 73% compared to the CRS group (Fig. 5G, H).

### Iron Hx stain of hippocampal tissue

**Fig. 6:**
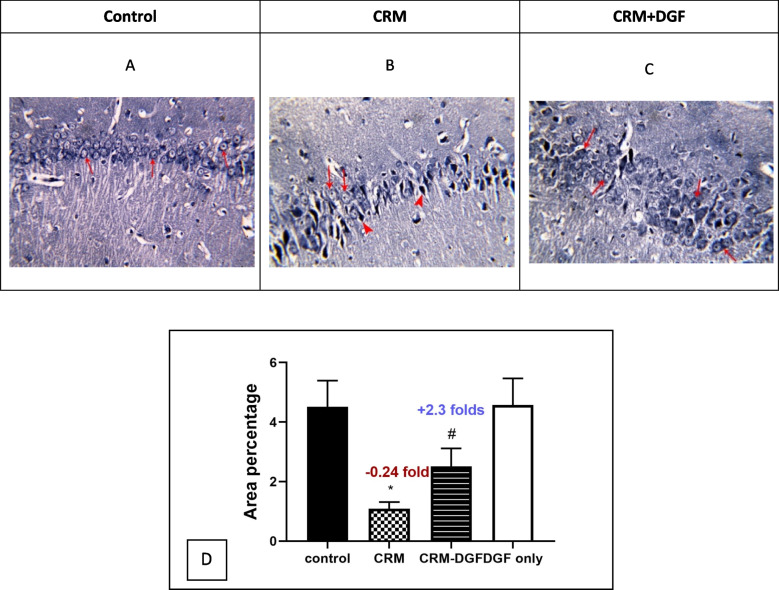
**A** Control group, **B** CRM group, **C** CRM + DGF group. **A** A photomicrograph of a section in the hippocampus (Iron Hx-stained sections) of a rat in the control group. Notice the thin, perinuclear, darkly stained area seen in the pyramidal cells (↑). **B** A photomicrograph of a section in the hippocampus of a rat in the CRM group. Many pyramidal cells are shrunken, deeply stained, and variable in size (filled triangle). Few pyramidal cells are seen with large, rounded nuclei. These cells have pale-stained perinuclear areas (↑). **C** A photomicrograph of a section in the hippocampus of a rat in the CRM + DGF group. Pyramidal cells are closely packed, most of them have central, rounded nuclei. Many pyramidal cells show a thin, perinuclear, darkly stained area (↑). **D** Area percentage of deeply stained perinuclear area in pyramidal cells in iron hematoxylin Iron Hx × 400

Iron Hx-stained sections of the hippocampus (Cornue Amonis) of rats in the control group and DGF only group showed a thin, perinuclear, darkly stained area seen in the pyramidal cells (Fig. 6A). CRM group showed few pyramidal cells are seen with large, rounded nuclei. These cells have pale-stained perinuclear areas (Fig. 6B). CRM + DGF group showed that many pyramidal cells have a thin, perinuclear, darkly stained area (Fig. 6C).

Chronic restraint stress of Wistar male rats significantly decreased area percentage (*P* < 0.0001) to 0.24-fold as compared to control group, while DGF treatment in CRS-DGF-administered group significantly increased area percentage (*P* < 0.0001) to 2.3-fold as compared to the CRS group (Fig. 6D).

### TEM of hippocampal tissue

**Fig. 7:**
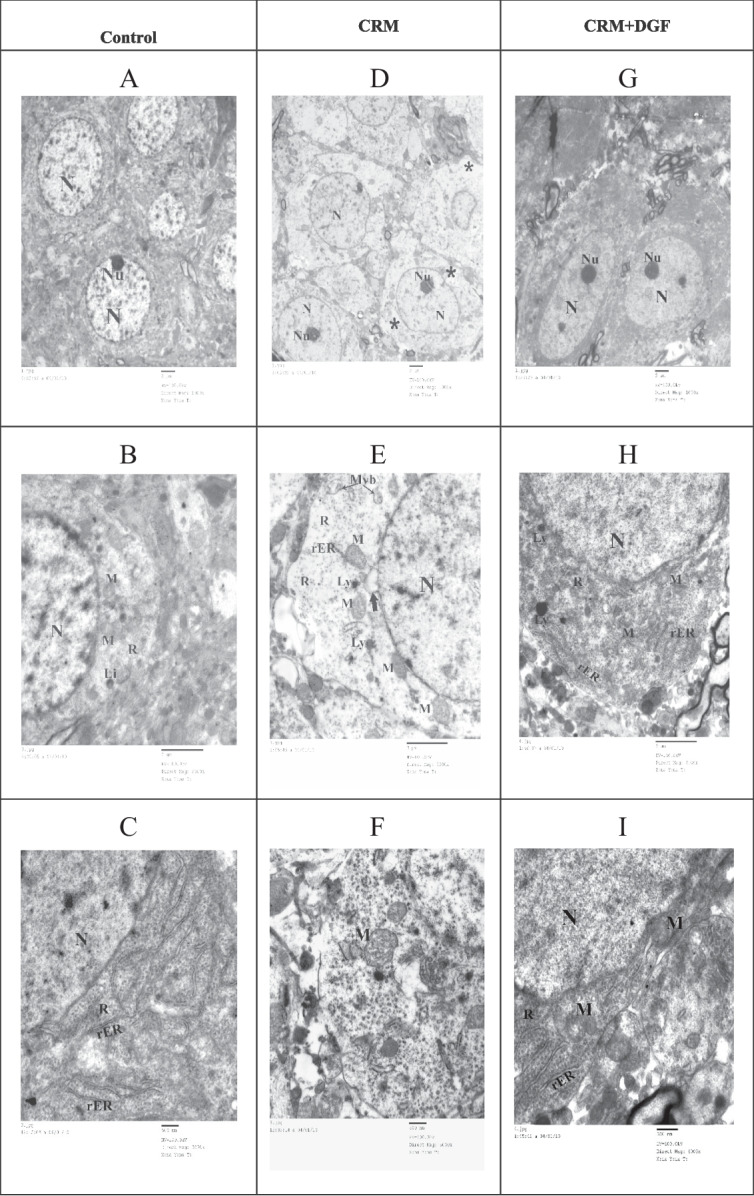
**A**, **B**, and **C** Electron micrographs of ultrathin sections in the hippocampus of rats in the control group. **A** Pyramidal cells are closely packed. Most of the pyramidal cells have euchromatic, central, rounded nuclei (N) with prominent nucleoli (Nu). **B** showing part of the nucleus (N) and cytoplasm of a pyramidal cell. Notice, the mitochondria (M) are nearly of the same shape and size. The cytoplasm contains abundant ribosomes (R). Few lysosomes (Ly) are seen. **C** showing part of the nucleus (N) and cytoplasm of a pyramidal cell. The cytoplasm contains abundant ribosomes (R) and prominent, well-developed cisternae of rER. **D**, **E**, **F**, and **G** Electron micrographs of ultrathin sections in the hippocampus of rat in the CRM group. **D** The pyramidal cells have central, rounded nuclei with abnormal chromatin distribution (N) and prominent nucleoli (Nu). The cytoplasm is seen electron lucent, vacuolated (*), and contains few widely separated organelles. **E** shows part of the nucleus (N) and cytoplasm of a pyramidal cell. Notice, the abnormal chromatin distribution in the nucleus. Many mitochondria are swollen (M), a mitochondrion is seen with broken cristae (thick arrow). The cytoplasm contains few ribosomes (R) and remnants of rER cisternae (rER). Few lysosomes (Ly) are seen. Notice the presence of multivesicular bodies (Mvb). **F** a pyramidal cell showing a swollen mitochondrion (M). **G**, **H**, and I Electron micrographs of ultrathin sections in the hippocampus of rats in the CRM + DGF group. **G** Pyramidal cells are closely packed. Most of the pyramidal cells have euchromatic, central, rounded nuclei (N), and prominent nucleoli (Nu). **H** part of the nucleus (N) and cytoplasm of a pyramidal cell showing mitochondria (M), abundant ribosomes (R), prominent, well-developed cisternae of rER, and few lysosomes (Ly). **I** showing part of the nucleus (N) and cytoplasm of a pyramidal cell. Mitochondria (M) are nearly of the same shape and size. The cytoplasm contains abundant ribosomes (R) and prominent, well-developed cisternae of rER. TEM: **A**, **D**, and **G** × 1000. **B**, **E**, and **H** × 3000. **C** and **F** × 5000 **I** × 6000

Transmission electron microscopic examination of ultrathin sections of the hippocampus of rats in the *control and DGF groups* showed closely packed pyramidal cells. Most of the pyramidal cells have euchromatic, central, rounded nuclei, with prominent nucleoli. The cytoplasm is densely packed with different organelles. The mitochondria are nearly of the same shape and size. The cytoplasm also contains abundant ribosomes, prominent, well-developed cisternae of rough endoplasmic reticulum (rER) and few lysosomes (Fig. 7A, B, C). However, *CRM group* showed that few pyramidal cells have central, rounded nuclei with prominent nucleoli. Their nuclei show abnormal chromatin distribution. The cytoplasm is seen electron lucent, with vacuoles and few widely separated organelles. Mitochondria are of variable shape and size. Many mitochondria are swollen, some mitochondria have broken cristae. The cytoplasm contains few ribosomes, remnants of rER cisternae and few lysosomes. The presence of multivesicular bodies is noticed (Fig. 7D, E, F).

*CRM* + *DGF-(treated) group* showed closely packed pyramidal cells. Most of the pyramidal cells have euchromatic, central, rounded nuclei with prominent nucleoli. The cytoplasm is densely packed with different organelles. Mitochondria are nearly of the same shape and size. The cytoplasm contains abundant ribosomes, well-developed cisternae of rER and few lysosomes (Fig. 7G, H, I).

## Discussion

Chronic stress and cognitive impairment (C.I.) are strongly interconnected, with a bidirectional relationship, where stress plays a key role in the onset and progression of neurodegenerative diseases, including Alzheimer’s disease (AD) (Armstrong et al. 2021). Conversely, neurodegenerative disorders lead to motor and cognitive dysfunctions that are inherently stressful and can disrupt brain circuits involved in stress responses (Mohammadi et al. 2022). Chronic stress results in prolonged elevation of glucocorticoid levels, which cause both functional and structural changes in the brain, particularly in the hippocampus. This region, a vital part of the limbic system, is essential for cognitive processes like learning and memory (Saeedi and Rashidy-Pour 2021).

We hypothesize that stress-induced behavioral and hormonal responses may contribute to the development of insulin resistance, which could, in turn, indirectly trigger neuropathological processes linked to the onset and progression of cognitive impairment (C.I.) and potentially lead to Alzheimer’s disease (AD) (Mravec et al. 2018). Insulin and insulin-like growth factor- 1 receptors are found in all cell types within the central nervous system, emphasizing the intricate role of insulin signaling in the brain (Arnold et al. 2018). Insulin resistance can compromise the permeability of the blood–brain barrier, which in turn can impair synaptic plasticity and contribute to cognitive dysfunction (Sędzikowska and Szablewski 2021).

Dapagliflozin (DGF), a selective and reversible SGLT2 inhibitor, is commonly used to treat type 2 diabetes (Dhillon 2019). The SGLT1 and SGLT2 receptors are widely expressed in the hippocampus, blood–brain barrier, endothelial cells, and cerebellum (Jurcovicova 2014). This distribution suggests that SGLT2 inhibitors could have neuroprotective effects, highlighting the promising use for DGF and other SGLT2 inhibitors in treating neurodegenerative diseases (Sa-Nguanmoo et al. 2017). The current study aimed to examine the effects of chronic DGF treatment on learning and memory deficits using the CRS model, further, to investigate the hypothesis that DGF may minimize chronic stress sequelae and C.I. either through its antioxidant effect, insulin sensitizing effect, or by improving mitochondrial dysfunction. The CRS model was chosen for its simplicity, cost-effectiveness, and non-invasive nature, as it does not cause physical harm to the animals (Ngoupaye et al. 2018).

One of the most important symptoms of C.I. is memory and learning difficulties (Levenson et al. 2014). Both Y maze and MWM tests are commonly used to assess memory and cognition deficits (Wolf et al. 2016). It was observed that CRS induced working memory deficits in Y maze testing manifested by a significant decline in spontaneous alteration ratio. Similar behavioral changes have been observed in previous studies (Kraeuter et al. 2019; Wei et al. 2022). Additionally, research on the cognitive effects of chronic social defeat stress demonstrated significant memory impairment and a notable reduction in the spontaneous alteration ratio (Wang et al. 2019). Additionally, deficits in learning and memory capacity were reflected by an increase in the latency to reach the platform in MWM test at the acquisition phase in rats which were exposed to CRS starting from day 3. These findings align with previous studies that have demonstrated memory and learning impairments in animals subjected to CRS (Jangra et al. 2016; Mehta et al. 2017; Wei et al. 2022).

Neurodegeneration was confirmed through histological examination of the CA1 area of the hippocampus, which showed pyramidal layer atrophy, irregularity, and reduced neuronal volume (Schaeffer et al. 2017). The pyramidal cells were widely spaced, irregularly arranged, and accompanied by vacuolated neutrophils. Most pyramidal cells were shrunken, deeply stained, and varied in size with condensed dark nuclei. Transmission electron microscopy (TEM) revealed cytoplasmic condensation and vacuolation in pyramidal cells, with few mitochondria, ribosomes, or lysosomes. Only remnants of rER with dilated cisternae were present, suggesting neurotransmitter synthesis deficiencies and the accumulation of toxic aggregates, a hallmark of neurodegenerative diseases (Paschen and Mengesdorf 2005). These findings ensure the occurrence of neurodegeneration induced by CRS (Kerchner et al. 2010 and Eslamizade et al. 2016). Also, multivesicular bodies (MVBs), a general neuronal stress indicator, are seen by electron microscopic examination as a compensatory mechanism of degeneration, yet it can be harmful as it allows transport of toxic material between cells and brain areas promoting the accumulation of this toxic materials in the extracellular space (Mathews and Levy 2019).

We hypothesize that this neurodegeneration may progress to AD, as TEM showed MVBs accumulating Aβ on their outer membranes before synaptic dystrophy. This Aβ leakage from MVBs is believed to be a key neurotoxic event in AD pathogenesis (Roos et al. 2021). Once Aβ accumulates in MVBs, it penetrates the membrane, entering the cytoplasm and causing cell death, extracellular Aβ deposition, and significant neuronal loss in the CA1 pyramidal cell layer, correlating with intraneuronal Aβ accumulation (Friedrich et al. 2010).

The progression of neurodegeneration was marked by amyloid deposits and tau hyperphosphorylation, which were biochemically assessed and found to be significantly elevated within neurons in the hippocampus of chronic stressed rats. The hippocampus, a primary target for glucocorticoid stress hormones, showed signs of aging, including dendritic atrophy, reduced neuronal density, impaired synaptic plasticity, and deficits in spatial learning (Sántha et al. 2012). Our results are consistent with previous studies that highlighted CRS-induced increases in soluble hyperphosphorylated tau in the hippocampus of rat brains (Carroll et al. 2011; Liu et al. 2019). Additionally, BDNF gene expression, an essential neuroprotective factor for synaptic plasticity and neuronal survival, was significantly disrupted in the stressed rats of our study, which is another critical factor ensuring the induction of C.I. by CRS and the occurrence of neurodegeneration.

CRS induces insulin resistance, characterized by hyperglycemia, hyperinsulinemia, and an increased HOMA-IR (Elbassuoni & Abdel Hafez 2019; Akinluyi et al. 2022). Insulin resistance (IR) accelerates AD progression by activating GSK- 3β, which promotes tau hyperphosphorylation, misfolding, and aggregation (Chatterjee et al. 2019). Additionally, Aβ peptides impair insulin signaling in the brain by competing with insulin or reducing receptor binding affinity (De La Monte 2012). Since Aβ and p-tau, key AD hallmarks, are influenced by IR and insulin signaling components (P-AKT and p-mTOR) (De La Monte and Tong 2014), this explains the strong link between IR and AD, as well as the cognitive impairment observed in stressed rats during behavioral assessment.

The current study revealed that DGF treatment prevented CRS associated memory deficits which is indicated by a significant increase in spontaneous alteration ratio in Y maze test. This finding is consistent with the study of El-Sahar et al. (2020) who showed improvement of behavioral dysfunction in a rat model of Huntington’s disease. Moreover, DGF treatment in our study limited stress induced memory and learning deficits by decreasing the latency to reach the platform in MWM at the acquisition phase in treated rats starting from day 3. It as well decreased the latency time to reach the target quadrant and increased the time spent inside it on the 6 th day in the probe trial. In accordance, previous animal studies showed improvement in MWM tasks after using DGF as a treatment in streptozotocin-induced diabetes (El-Safty et al. 2022). Another study stated that DGF can markedly improve the cognitive dysfunction in AD-induced rat model (Hazar-Yavuz et al. 2022). Improvement in C.I. by DGF has been ensured by histological examination of CA1 area which showed improvement in pyramidal cell layer thickness, increasing the number of normal pyramidal cells having large, rounded, central, and vesicular nuclei with prominent nucleoli. In addition, by TEM, most of the pyramidal cells after DGF treatment have euchromatic, central, rounded nuclei, and densely packed cytoplasm with different organelles. Abundant ribosomes and prominent; slightly dilated cisternae of rER are present with no MVBs. These findings are all signs of neuronal and plasticity improvement (Gasparova et al. 2014; Hosseini-Sharifabad and Esfandiari 2015), which further confirms the neuroprotective effect DGF and C.I. improvement induced by CRS.

Chronic restraint stress (CRS) significantly reduced BDNF levels, as noted in previous research (Shin et al. 2019; Wang et al. 2020). This reduction may be linked to CRS-induced decreases in either the expression or release of BDNF (Ju et al. 2022) or it could result from elevated glucocorticoid (GC) levels caused by CRS, which impair glucocorticoid receptor (GR)–tropomyosin receptor kinase B (TrkB) interactions, disrupting BDNF functions in the brain (Chiba et al. 2012). Biochemically, DGF effectively reversed the drop in BDNF mRNA expression, thereby promoting the BDNF signaling pathway, enhancing neurogenesis, and positively influencing cognitive function in the rats. Additionally, DGF reduced Aβ and p-tau levels compared to the CRS group and even lowered Aβ levels compared to the control rats. These results suggest that DGF can mitigate neurodegenerative changes and slow Alzheimer’s disease (AD) progression associated with CRS. While similar studies are limited, a study on a diabetes-induced AD rat model showed comparable outcomes, indicating that DGF has effects similar to the anticholinesterase inhibitor rivastigmine, a drug commonly used in AD treatment. Furthermore, various studies have explored the role of BDNF in neurodegenerative diseases and its associated mechanisms (Kang et al. 2022).

Corticosterone (CORT) is the maestro of stress as it mediates most of its deleterious effects. Exposure to CRS significantly elevated serum CORT levels as it is considered the main glucocorticoid involved in regulation of stress responses in rodents as it activates the hypothalamic–pituitary–adrenal (HPA) axis and increases the release of glucocorticoids (Vyas et al. 2016). Furthermore, CRS downregulates the expression of glucocorticoid receptors (Chiba et al. 2012) and leads to sustained increased CORT exposure which becomes maladaptive causing wide range of disease states (Biddie et al. 2012).

One of the suspected mechanisms through which CRS impairs cognition is its deteriorating effect on glucose homeostasis. CORT exposure promotes gluconeogenesis, inhibits insulin production, and impairs insulin signaling (Joseph and Golden 2017). Insulin resistance is a critical cause of C.I. as it changes neurite outgrowth and disrupts neurotransmitter uptake and release (Arnold et al. 2018). Additionally, CORT exposure impairs neuronal plasticity, decreases synapses’ density, and results in glucocorticoid resistance in the brain (Merkulov et al. 2017). CORT can activate phosphoinositide 3 kinase (PI3 K)/Akt signaling by binding to glucocorticoid receptor and β-adrenergic receptors stimulation secondary to adrenergic stimulation, finally disrupting the insulin signaling pathway (Zhang et al. 2021).

P-AKT and p-mTOR are two critical components in insulin signaling pathway which have an important role in survival of different cell types (Zhang et al. 2019). Elevations in p-AKT and p-mTOR levels were observed in chronic restraint stressed rats compared to control rats. The mTORC1 signaling pathway is important in mediating synaptic loss triggered by chronic stress in both the ventral and dorsal hippocampus (Luo et al. 2021). Chronic CORT elevation also affects mTOR pathway directly, which is essential for translational control and has long-term consequences for the plasticity of different brain circuits (Polman et al. 2012). Continuous mTOR upregulation is linked to amyloid β, tau aggregation, and hyperphosphorylation in AD (Van Skike and Galvan 2018). In contrary to our results, Akt-mTOR signaling pathway was inactivated in chronic mild stress (CMS) model as well as its downstream effectors (Zhu et al 2019). Also, reduction in the level of phosphorylation of mTOR protein and p-AKT level were observed under CRS (Yan et al. 2023).

Sustained CORT exposure in chronic stress, along with catecholamines, induces metabolic alterations such as glucotoxicity and lipotoxicity, leading to oxidative stress and inflammation (Bagheri et al. 2021). This increases oxidative markers and reactive oxygen species in the cortex and hippocampus (Gerecke et al. 2013). Oxidative stress also promotes Aβ and tau aggregation, phosphorylation, and polymerization (Huang et al. 2016). Moreover, IR causes oxidative damage, protein aggregation, ATP depletion, mitochondrial ROS (mROS) overproduction, and redox imbalance (Verdile et al. 2015; Żukowski et al. 2018). This data was confirmed in our work as CRS elicited significant increase in MDA with significant reduction in GSH which ensured the presence of oxidative stress. Our study supports past studies which showed that different stress models reduce antioxidant enzyme activity and enhance free radical generation (Salehi et al. 2018, Dong et al. 2019 and Yisireyili et al. 2020).

Also, we have deduced from our work that mitochondrial function was greatly impaired by CRS, which is indicated by ATP depletion and reduction of CCO activity compared to the control group. Consistent with our findings, Suwanjang et al. (2021) also found that chronic stress induced mitochondrial dysfunction and neuronal cell death. Mitochondrial affection in our study was evident by TEM which revealed significantly reduced mitochondria in degenerated neurons which suffers synaptic pathology. Mitochondrial alterations were prominent whenever Golgi apparatus fragmentation exists (Macdonald et al. 2018 and Takeda et al. 2021). The loss of structural and functional mitochondrial integrity is causally associated with energy metabolism impairment and oxidative stress enhancement in AD (Wang et al. 2020).

Chronic stress, oxidative damage, and elevated mROS levels lead to brain mitochondrial dysfunction, which contributes to neuronal apoptosis, impaired synaptic plasticity, cerebral degeneration, and cognitive decline in the insulin-resistant brain (Maciejczyk et al. 2019). Our findings are consistent with Toma et al. (2017), who reported that CRS reduced CCO activity in both the prefrontal cortex and hippocampus. Based on this, we link CRS and subsequent insulin resistance to neuronal oxidative stress, which negatively impacts Aβ and tau, contributing to cognitive impairment and AD pathophysiology.

Interestingly, chronic DGF treatment decreased serum corticosterone levels significantly and limited its elevation when compared to control rats, thus decreasing the deleterious effects of CORT elevation including obesity, metabolic disturbances, IR, and C.I. Further, DGF reversed the increase in body weight induced by stress as it was proved to reduce total body weight by reducing body fat mass, visceral, and subcutaneous adipose tissue in T2DM (Bolinder et al 2012). This is consistent with previous research which showed that DGF is effective in decreasing body weight and improving glycemic control (Lazzaroni et al. 2021).

DGF significantly corrected CRS-induced metabolic disturbances by lowering serum glucose levels and improving insulin sensitivity, as indicated by a decrease in HOMA-IR, without affecting serum insulin levels. As an anti-diabetic drug, DGF improves insulin resistance and controls blood glucose without increasing insulin secretion (Ng et al. 2021; Fakhrolmobasheri et al. 2023). Additionally, DGF enhanced insulin signaling by reducing p-AKT and p-mTOR levels. Our findings align with those of Stanciu et al. (2021) and Ibrahim et al. (2022).

DGF improves insulin resistance through several mechanisms, including normalizing hyperglycemia to near-physiological levels, reducing the risk of glucotoxicity, promoting caloric expenditure and weight loss, decreasing oxidative stress and inflammation, and enhancing beta cell function by altering the pathophysiological pathways involved in islet cell death (Yaribeygi et al. 2020; Tanday et al. 2022). The improvement of insulin resistance (IR) and its detrimental effects on the brain, along with enhanced insulin signaling by DGF, confirmed our initial hypothesis that DGF improves cognitive function and enhances learning and memory in treated rats.

We found that DGF administration ameliorated oxidative stress as it significantly increased GSH levels and decreased MDA levels. It also improved mitochondrial function in stressed rats by enhancing ATP synthesis and CCO activity. Previous studies Kingir et al. (2019) and Hazem et al. (2022) supports our finding although the exact antioxidant mechanism of DGF remains unclear. A recent hypothesis suggests that DGF’s antioxidant properties may be linked to its ability to lower uric acid and insulin levels (La Grotta et al. 2022). Improvement of mitochondrial function, reduced ROS production, and altered Ca^2+^ dynamics are also proposed mechanisms (Zaibi et al. 2021; Li et al. 2022). Additionally, DGF restored mitochondrial membrane potential, energy metabolism, mitochondrial viability, biogenesis, and structural injury (He et al. 2022). Histological TEM examination confirmed these effects, showing improved mitochondrial number and shape following DGF treatment.

## Conclusion

This study supports the biological link between chronic stress, insulin resistance and C.I. CRS induced learning and memory impairment symptoms (decreased spontaneous alteration ratio in Y maze test and decreasing latency with increasing time spent in target quadrant in MWM test) in addition to neurodegenerative changes in CA1 of hippocampus with metabolic disturbances (increased glucose, cortisol, and HOMA-IR with decreased insulin secretion), impairing insulin signaling pathway. Dapagliflozin through its insulin sensitizing effect improves insulin signaling by enhancement of BDNF gene expression along with decreasing Akt and mTOR, its antioxidant actions via reducing MDA and increasing GSH, mitochondrial function improvement by increasing ATP synthesis and enhances CCO activity expression in hippocampus and has promising effects on ameliorating cognitive deficits and neurodegeneration.

## Limitations

Since our research was conducted on experimental animals, the results may not directly translate to humans. We focused mainly on oxidative stress, insulin resistance, and mitochondrial dysfunction, but other factors like neuroinflammation might also play a role in cognitive deficit and need further exploration. Moreover, the treatment duration was relatively short, so the long-term effects of dapagliflozin on brain function is yet not known, and potential differences between male and female subjects have not been assessed, which could be important for understanding how broadly these findings apply. Future studies should aim to address these gaps by extending treatment periods, exploring additional mechanisms, and eventually testing these effects in clinical settings.

## Recommendations

Based on the findings of this study, dapagliflozin could offer a promising therapeutic approach for addressing chronic stress-induced learning and memory impairments, as well as its potential to limit neurodegeneration. However, comprehensive clinical studies are necessary to evaluate the safety and efficacy of dapagliflozin in individuals experiencing chronic stress. In addition, further experimental research should explore the different biochemical and molecular pathways through which dapagliflozin can enhance cognitive function in chronic stress. Identifying these mechanisms will be crucial in optimizing its use as a treatment for stress-related cognitive impairments.

## Data Availability

All source data for this work (or generated in this study) are available upon reasonable request.
